# Tuberculosis and Syphilis

**Published:** 1907-09-21

**Authors:** 


### TUBERCULOSIS AND SYPHILIS.

The symbiosis of disease has received very little attention in this country—that is to say, the interaction of one disease upon another when the two coexist. Dr. C. F. Marshall contributes an interesting and very necessary article upon tuberculosis and syphilis to a recent number of the "British Journal of Tuberculosis." He draws attention to the fact that there are affections regarded as tuberculous in which the tubercle bacillus is rarely found; that there are also affections often looked upon as tuberculous which are really syphilitic; and that there are hybrid affections which are combinations of syphilis and tubercle, though they tend towards one or the other type of disease, according as there is a predominance of the one or the other factor. The first two classes can be separated by a careful study of the signs and symptoms with which they are associated; the last it is impossible to differentiate as a general rule. Dr. Emile Sergent, of Paris, has done good service to the subject of this particular form of symbiosis in his book on "Syphilis et Tuberculose," which was issued at the beginning of the year. It contains a careful study of difficult cases which have been for the most part wholly misinterpreted by the medical attendant owing to the prevailing ignorance of the idea that it is possible to have a tuberculate of syphilis or a syphilate of tubercle. This combination of tubercle and syphilis comes under the notice of several branches of the profession with tolerable frequency. It is found in the respiratory system, in the bones and joints, in the skin, and in the lymphatic glands. A tuberculous patient sometimes acquires syphilis; more often a patient who is born syphilitic becomes tuberculous, for his tissues are inherently weakened, and he falls an easy prey to microbic infection. The latter cases are by far the most difficult to recognise. Those which it is tolerably easy to recognise are the children who present well-marked signs of inherited syphilis, or where, and perhaps by accident, the administration of mercury has worked miracles in amending a long-standing disease which was always looked upon as tuberculous. It is indeed doubtful whether the reputation of grey powder in the treatment of the tuberculous disease of children is not almost entirely due to its action as an anti-syphilitic rather than to any specific effect which it exercises upon the products of tuberculous infection. The really difficult cases are those in which tuberculosis attacks late in middle life the joints of a patient who was born syphilitic; in other words, where senile tuberculosis is grafted upon a syphilitic inheritance. Such a patient denies with perfect truthfulness that he has ever acquired syphilis or suffered from any form of venereal disease. He can point to healthy children, and his wife has had no miscarriages or any illness which will invalidate the truth of his story. He is ignorant of any childish ailments, and he has long outlived those who could throw any light upon them or upon the illnesses of the preceding generation. A reference to the ancestral death certificates affords no assistance, for they are either cautiously worded or so vague as to be unintelligible. A skiagram may show at once the nature of the disease if the bone alone is affected, but it is useless in joint lesions. When the joints are affected the diagnosis can only be made by sections of the synovial membranes to show the thickened walls of the blood-vessels and lymphatics. The articular cartilage, too, shows changes both to the naked eye and under the microscope which prove that it is the seat of neither a syphilitic nor a tuberculous lesion. But the inquiry as to the exact diagnosis is barren as regards treatment in these cases. Mercury is ineffectual; the iodides do little more than reduce the swelling for a short time, and cod-liver oil is useless. Rest, massage, and the adoption of every possible means to improve the general health of the patient alone do good. But when the synovial membrane and the cartilages of a large joint are affected by tubercle grafted on a syphilitic soil, amputation is usually the sole resource at the command of the surgeon, and *amputatio opprobrium chirurgi* is an adage of many years' standing. This confession of failure is gradually disappearing, for the chief work of a surgeon no longer consists in the dismemberment of his patients as is still popularly supposed. In reality amputation is a comparatively rare operation, and a student may go through at least one course of dressing without seeing it performed. Still the care of a surgeon is not at an end when he has removed a tuberculo-syphilitic joint, since the tissues are so thoroughly diseased and the vessels are in such an unhealthy state that secondary hæmorrhage is of somewhat common occurrence, and a phagedænic inflammation is by no means rare.

